# Educational inequalities in epilepsy mortality in the Baltic countries and Finland in 2000–2015

**DOI:** 10.1038/s41598-022-08456-x

**Published:** 2022-03-17

**Authors:** Andrew Stickley, Aidan Neligan, Aleksei Baburin, Domantas Jasilionis, Juris Krumins, Pekka Martikainen, Naoki Kondo, Tomiki Sumiyoshi, Jae Il Shin, Hans Oh, Kyle Waldman, Mall Leinsalu

**Affiliations:** 1 Stockholm Centre for Health and Social Change, Södertörn University, 141 89 Huddinge, Sweden; 2 Department of Social Epidemiology, Graduate School of Medicine and School of Public Health, Kyoto University, Kyoto, Japan; 3 Department of Preventive Intervention for Psychiatric Disorders, National Institute of Mental Health, National Center of Neurology and Psychiatry, Tokyo, Japan; 4 Homerton University Hospital NHS Foundation Trust, Homerton Row, London, E9 6SR UK; 5 DCEE, UCL Queen Square Institute of Neurology, Queen Square, London, WC1N 3BG UK; 6 Department of Epidemiology and Biostatistics, National Institute for Health Development, Tallinn, Estonia; 7 Max Planck Institute for Demographic Research, Rostock, Germany; 8 Demographic Research Centre, Vytautas Magnus University, Kaunas, Lithuania; 9 Demography Unit, Faculty of Business, Management and Economics, University of Latvia, Riga, Latvia; 10 Population Research Unit, University of Helsinki, Helsinki, Finland; 11 Department of Public Health Sciences, Stockholm University, Stockholm, Sweden; 12 Department of Pediatrics, Yonsei University College of Medicine, Yonsei-ro 50, Seodaemun-gu, Seoul, Korea; 13 Suzanne Dworak-Peck School of Social Work, University of Southern California, 1149 South Hill Street suite 1422, Los Angeles, CA 90015 USA; 14 Department of Sociology, Harvard University, Cambridge, MA USA

**Keywords:** Neurological disorders, Epilepsy, Epidemiology

## Abstract

Little is known about socioeconomic differences in epilepsy mortality. This study examined educational inequalities in epilepsy mortality in the general population in the Baltic countries and Finland in 2000–2015. Education-specific mortality estimates for individuals aged 30–74 in Estonia, Latvia and Lithuania were obtained from census-linked mortality datasets while data for Finland came from the register-based population and death data file of Statistics Finland. Trends and educational inequalities in epilepsy mortality were assessed using age-standardised mortality rates (ASMRs) per 100,000 person years and age-adjusted mortality rate ratios (RRs) calculated using Poisson regression. ASMRs were higher in men than women in all countries. ASMRs reduced in 2000–2015 among all men and women except for Finnish women. Among men, an inverse educational gradient in epilepsy mortality in 2000–2007 widened in 2008–2015 with ASMRs falling among high and mid educated men in all countries but increasing among low educated men in three countries. An inverse educational gradient in female mortality remained in all countries throughout 2000–2015. Although epilepsy mortality fell in the Baltic countries and Finland (men only) in 2000–2015, this masked a clear inverse educational gradient in mortality that became steeper across the period.

## Introduction

Epilepsy is a significant cause of mortality. In 2016, there were over 126,000 epilepsy-related deaths worldwide^1^. Partly this reflects the fact that death rates are elevated in people with epilepsy (PWE). Specifically, a recent review of population-based studies in high-income countries by the International League Against Epilepsy (ILAE) Commission on Epidemiology found standardised mortality ratios (SMRs) ranging from 1.6 to 3.0 among PWE compared to the general population^2^, while a corresponding study on low- and middle-income countries reported an even wider range of figures, from 1.3 to 7.2^3^. Despite an overall reduction in global epilepsy age-standardised mortality rates of almost 25% between 1990 and 2016, some countries nevertheless experienced large increases in their death rates during this period^1^, while other research has also highlighted that epilepsy mortality rates can differ consistently between populations in different countries over time^4^. This supports the recent call for epilepsy-related mortality to be regarded as a public health priority^5^.

Despite the fact that epilepsy is seemingly linked to an increased mortality risk, many factors associated with this phenomenon remain under-researched. For example, as yet, there has been surprisingly little focus on socioeconomic variation in epilepsy deaths, even though low socioeconomic status has been linked to risk factors for epilepsy (e.g. head trauma, brain neoplasms) that might also result in higher mortality in PWE^6^. Importantly, there is some evidence that epilepsy mortality is associated with economic disadvantage and that a socioeconomic gradient might exist in epilepsy deaths. A retrospective cohort study of PWE in South Carolina showed that living in areas with low median household income was associated with an increased mortality risk in 2000–2013^7^. A recent review study on epilepsy deaths has also underlined the potential for socioeconomic variation in epilepsy mortality by noting that there was a nearly threefold difference in age-standardised epilepsy mortality rates between the most and least deprived neighbourhood deciles in England (13.1 vs. 5.1 per 100,000)^8^.

Against this backdrop, the current study will examine if there were socioeconomic differences, as assessed by differences in educational attainment, in epilepsy mortality among the general adult population in the Baltic countries (Estonia, Latvia, Lithuania) and Finland in 2000–2015. The Baltic countries have somewhat differing healthcare systems. Estonia and Lithuania have social health insurance systems underpinned by employment-based contributions with government funding for other non-employed groups, while in Latvia there is a national healthcare system funded through income tax and general revenues^9,10^. Although these systems provide extensive medical coverage (from over 90% of the population in Estonia and Lithuania to universal coverage in Latvia), high user charges (in Latvia) and substantial out-of-pocket payments can limit access to healthcare services, especially in disadvantaged groups^9,11^. Comparatively little is known about the epidemiology of epilepsy in the Baltic countries^12^. However, there is some indirect indication that socioeconomic differences might be important for epilepsy mortality in these countries. Research from Estonia has shown, for example, that in a setting where epilepsy may be undertreated^13^, the level of education is linked to employment status among PWE, which in turn (i.e. underemployment/unemployment) may be associated with epilepsy severity^14^. This may be of relevance as other research has linked more seizures to an increased risk for sudden unexpected death in epilepsy (SUDEP)^15^. The results will be compared to Finland, a neighbouring Nordic welfare state, that has three times higher per capita health care spending and a much lower share of out-of-pocket payments^11^.

Thus, building on and extending several recent studies that have examined epilepsy mortality in the general population (i.e. among all individuals irrespective of diagnostic status)^4,16,17^ this study has two main aims: (i) to examine changes in epilepsy mortality in the general population in the Baltic counties and Finland in 2000–2015; (ii) to determine if there are educational inequalities in epilepsy mortality in these countries and if there are, whether there have been any changes in them across the study period. Establishing whether there are educational inequalities in epilepsy mortality in these countries may be important for future attempts to reduce these deaths.

## Methods

### Mortality data

Data for Estonia, Latvia and Lithuania were obtained from census-linked mortality datasets based on the linkages between the population censuses in 2000 (2001 in Lithuania) and 2011 and death records involving all permanent residents. The censuses in the Baltic countries combined traditional survey-based enumeration (the share of coverage ranged from 91% in Latvia to 98% in Estonia) and register-based enumeration^18^. The register-based data did not include information about socioeconomic status (educational attainment) and were therefore excluded from the analysis. All individuals were followed from the census date until the date of death or emigration, or until the end of the follow-up period. The date and cause of death were linked from national mortality registries. All data linkages were performed by National Statistical Offices. Corresponding data for Finland were obtained from the register-based education-specific population and death data file of Statistics Finland covering the permanent total population. Data were originally organised into four sub-periods: 2000–2003, 2004–2007, 2008–2011 and 2012–2015. To ensure that a complete educational history was obtained and to achieve optimal accuracy of cause of death ascertainment, this study focuses on those aged 30–74 years. Population exposures were estimated by adding up the number of person years lived by individuals within each 5-year interval age group in a given period. Deaths were allocated to age intervals using the age at death. Data were anonymised and aggregated into multidimensional frequency tables combining deaths and population exposures split by study periods and sociodemographic variables before they were delivered for research purposes. As this study used secondary anonymised data ethical permission was not required.

### Measures

Following the lead of earlier studies in the general population^4,19^, all deaths where the underlying cause was classified as G40 (Epilepsy) or G41 (Status epilepticus) using the 10th revision of the International Classification of Diseases (ICD-10)^20^ were considered as deaths from epilepsy (Appendix 1). Sociodemographic data are census-based and were coded by the statistical offices following a common study protocol. Socioeconomic status was assessed using data on educational level, which was categorised using the International Standard Classification of Education (ISCED) 2011^21^. *Low* education refers to primary and lower secondary education (ISCED categories 0–2), *middle (mid)* education includes upper secondary and post-secondary non-tertiary education (categories 3–4), and *high* education covers tertiary education (categories 5–8). The percentage of missing values for education was low (0–0.7%) and these cases were additionally excluded from the analysis (Table 1).

**Table 1:** Characteristics of the study populations and age-standardised mortality rates per 100,000 person years for epilepsy in 2000–2015 in the 30–74 age group.

Sex	Country	Period	Deaths	Person	Educational level				ASMR (95% CI)	Change	*p*-value
				Years	High	Middle	Low	Missing		2008–2015 vs.	
			*N*	*N*	%	%	%	%		2000–2007	
Men	Finland	2000–2007	407	11,597,283	26.8	39.3	33.9	0.0	3.4 (3.1–3.8)		
		2008–2015	351	11,963,418	28.7	44.5	26.9	0.0	2.8 (2.5–3.1)		
										− 0.6	0.008
	Estonia	2000–2007	234	2,540,646	25.4	48.7	25.2	0.7	9.4 (8.2–10.6)		
		2008–2015	197	2,634,549	27.6	51.2	20.7	0.5	7.5 (6.5–8.6)		
										− 1.9	0.023
	Latvia	2000–2007	323	3,989,498	15.3	58.2	25.9	0.6	8.2 (7.3–9.1)		
		2008–2015	200	3,927,536	18.3	62.4	19.1	0.3	5.1 (4.4–5.8)		
										− 3.1	< 0.001
	Lithuania	2001–2007	418	5,836,369	16.3	59.8	23.3	0.5	7.2 (6.5–7.9)		
		2008–2015	386	6,463,171	19.3	61.0	19.4	0.3	5.9 (5.3–6.5)		
										− 1.3	0.006
Women	Finland	2000–2007	181	11,834,081	31.3	36.2	32.5	0.0	1.5 (1.3–1.7)		
		2008–2015	190	12,111,012	36.5	40.1	23.4	0.0	1.5 (1.3–1.7)		
										0.0	0.944
	Estonia	2000–2007	62	3,138,593	34.7	44.9	19.9	0.5	2.0 (1.5–2.5)		
		2008–2015	39	3,132,532	40.0	46.2	13.4	0.3	1.3 (0.9–1.7)		
										− 0.7	0.029
	Latvia	2000–2007	57	5,073,222	19.0	58.7	21.9	0.4	1.2 (0.9–1.5)		
		2008–2015	47	4,854,352	26.1	60.0	13.7	0.2	1.0 (0.7–1.2)		
										− 0.2	0.234
	Lithuania	2001–2007	89	7,018,533	19.0	59.3	21.2	0.5	1.3 (1.0–1.6)		
		2008–2015	94	7,677,380	24.7	60.4	14.6	0.3	1.2 (0.9–1.4)		
										− 0.1	0.549

The follow-up in the 1st period started from the census date in the Baltic countries, i.e. 31.03.2000 in Estonia, 1.03.2000 in Latvia, and 6.04.2001 in Lithuania; in 2008 the follow-up started on January 1; the follow-up ended on December 31 in the respective periods.

*ASMR* age-standardised mortality rate per 100,000 person years, *CI* confidence interval.

### Statistical analysis

To increase the statistical power we combined data into two sub-periods: 2000–2007 and 2008–2015. This cutoff point coincided with the end of a period of strong economic growth that was followed by a sharp, although relatively short-term recession after 2008^22^. Age-standardised mortality rates (ASMRs) per 100,000 person years were calculated using the European Standard Population^23^. Statistical testing of the change in the ASMRs between study periods was performed and exact p-values were included in the tables; the level of statistical significance was set at *p* < 0.05. Educational inequalities in epilepsy mortality were assessed by age-adjusted mortality rate ratios (RR) calculated using Poisson regression. ASMRs and RRs are presented together with 95% confidence intervals (CI). To assess the magnitude and direction of the potential bias related to the exclusion of register-based data from census records in the Baltic countries we performed a sensitivity analysis for Latvia comparing ASMRs for epilepsy mortality while excluding and including register-based data. Statistical analyses were performed using SPSS Statistics for Windows, version 26.0 (IBM Corp. 2019) and STATA 14.2 (Stata Corp., College Station, Texas, USA).

## Results

There were 3275 epilepsy deaths in the four countries in 2000–2015 and approximately 103.8 million person years of follow-up. Among men, ASMRs were much higher in the Baltic countries than in Finland in 2000–2007 ranging from 7.2 (95%: 6.5–7.9) (Lithuania) to 9.4 (95% CI: 8.2–10.6) (Estonia) per 100,000. Male epilepsy deaths decreased in all countries from 2000–2007 to 2008–2015 with the reduction in ASMRs ranging from -0.6 per 100,000 in Finland to -3.1 in Latvia (Table 1). In all countries the reduction in male ASMRs was statistically significant. Among women ASMRs were lower than for men and similar across the four countries, ranging from 1.2 (95% CI: 0.9–1.5) (Latvia) to 2.0 (95% CI: 1.5–2.5) (Estonia) per 100,000 in 2000–2007. A reduction occurred in female epilepsy ASMRs in all countries except Finland across the study period ranging from -0.1 (Lithuania) to -0.7 (Estonia) per 100,000 but was only statistically significant in Estonia.

Among men a clear educational gradient was observed in epilepsy mortality in all of the countries in 2000–2007 (Table 2). Among the high educated, epilepsy ASMRs ranged from 1.5 (95% CI: 1.0–1.9) (Finland) to 5.1 (95% CI: 3.4–6.9) (Estonia) per 100,000, while the corresponding figures for the mid educated were 3.4 (95% CI: 2.8–3.9) (Finland) to 8.2 (95% CI: 6.5–9.9) (Estonia), and for the low educated ASMRs ranged from 5.7 (95% CI: 4.9–6.5) (Finland) to 18.5 (95% CI: 15.2–21.7) (Latvia) per 100,000. Compared to the high educated, those with mid education had mortality rate ratios ranging from 1.6 (95% CI: 1.1–2.4) (Estonia) to 3.1 (95% CI: 1.8–5.4) (Latvia), while the corresponding figures for the low educated were 3.3 (95% CI: 2.2–4.9) (Estonia) to 8.3 (95% CI: 4.7–14.7) (Latvia). From 2000–2007 to 2008–2015 a reduction was observed in epilepsy ASMRs among all groups except for in three of the low educated groups where there was an increase of 0.4 (Finland) to 1.1 (Lithuania) per 100,000. The reduction was significant for the high educated in Finland and Estonia, for the mid educated in all countries but Estonia, and for the low educated in Latvia. In all other groups the changes were not statistically significant. These changes resulted in the educational gradient widening among all groups in all countries. Thus, compared to high educated individuals, those with mid education had rate ratios ranging from 2.1 (95% CI: 1.4–3.4) (Estonia) to 4.3 (95% CI: 2.1–8.8) (Latvia), while for the low educated, the figures were 5.5 (95% CI: 3.5–8.7) (Estonia) to 10.1 (95% CI: 4.8–21.0) (Latvia).

**Table 2:** Age-standardised mortality rates per 100,000 person years and mortality rate ratios by educational level for epilepsy in 2000–2015 among men in the 30–74 age group.

Country	Educational level	ASMR (95% CI)		Change 2008–2015 vs. 2000–2007	*p* − value	Rate ratio (95% CI)	
		2000–2007	2008–2015			2000–2007	2008–2015
Finland	High	1.5 (1.0–1.9)	0.8 (0.5–1.0)	− 0.7	0.007	1	1
	Mid	3.4 (2.8–3.9)	2.5 (2.1–2.9)	− 0.9	0.012	2.4 (1.7–3.4)	3.0 (2.0–4.5)
	Low	5.7 (4.9–6.5)	6.1 (5.1–7.1)	0.4	0.542	3.8 (2.7–5.2)	7.1 (4.8–10.5)
Estonia	High	5.1 (3.4–6.9)	3.0 (1.8–4.2)	− 2.1	0.046	1	1
	Mid	8.2 (6.5–9.9)	6.7 (5.3–8.1)	− 1.5	0.171	1.6 (1.1–2.4)	2.1 (1.4–3.4)
	Low	17.0 (13.3–20.7)	17.8 (13.6–22.0)	0.8	0.779	3.3 (2.2–4.9)	5.5 (3.5–8.7)
Latvia	High	2.0 (0.9–3.1)	1.1 (0.3–1.9)	− 0.9	0.194	1	1
	Mid	6.7 (5.6–7.8)	4.8 (3.9–5.6)	− 1.9	0.006	3.1 (1.8–5.4)	4.3 (2.1–8.8)
	Low	18.5 (15.2–21.7)	11.3 (8.4–14.2)	− 7.2	0.026	8.3 (4.7–14.7)	10.1 (4.8–21.0)
Lithuania	High	2.6 (1.5–3.6)	1.6 (0.9–2.3)	− 1.0	0.124	1	1
	Mid	6.9 (6.0–7.8)	5.3 (4.5–6.0)	− 1.6	0.007	2.6 (1.7–4.0)	3.2 (2.0–5.1)
	Low	14.6 (11.9–17.3)	15.7 (12.9–18.5)	1.1	0.582	5.5 (3.6–8.4)	9.0 (5.6–14.5)

*ASMR* age-standardised mortality rate per 100,000 person years, *CI* confidence interval.

Among women the results were more variable (Table 3). In 2000–2007, low educated women had higher epilepsy ASMRs compared with the high educated in all countries. Among the high educated, ASMRs ranged from 0.2 (95% CI: 0.0–0.4) (Latvia) to 1.5 (95% CI: 0.8–2.2) (Estonia) per 100,000, for the mid educated from 0.8 (95% CI: 0.5–1.2) (Latvia) to 1.5 (95% CI: 0.9–2.1) (Estonia) and for the low educated from 3.4 (95% CI: 2.6–4.1) (Finland) to 7.1 (95% CI: 3.8–10.4) (Estonia) per 100,000. Rate ratios for the low educated ranged from 3.3 (95% CI: 1.7–6.5) (Estonia) to 25.4 (95% CI: 6.0–107.8) (Latvia) compared to the high educated, while the corresponding figures for the mid educated varied from 1.0 (95% CI: 0.5–2.0) (Estonia) to 4.0 (95% CI: 1.0–17.0) (Latvia). In Finland there was an increase in ASMRs across all educational groups in 2008–2015 compared to 2000–2007 although there was little change in the mortality rate ratios in this period. In contrast, the results varied for women by educational group in the Baltic countries where ASMRs decreased or remained the same among the high and mid educated (in Latvia also among the low educated) but increased among the low educated in Estonia and Lithuania. Except for high educated Estonian women, the changes in ASMRs were not statistically significant. These changes resulted in epilepsy mortality rate ratios rising in the mid and low educated in Estonia and falling in these groups in Latvia; in Lithuania the rate ratio increased for the low educated but decreased for the mid educated. Noticeably, in 2008–2015 the mortality rate ratios of low educated women in the Baltic countries were especially elevated ranging from 11.7 (95% CI: 5.8–23.9) in Lithuania to 19.2 (95% CI: 5.6–66.0) in Latvia.

**Table 3:** Age-standardised mortality rates per 100,000 person years and mortality rate ratios by educational level for epilepsy in 2000–2015 among women in the 30–74 age group.

Country	Educational level	ASMR (95% CI)		Change 2008–2015 vs. 2000–2007	*p* − value	Rate ratio (95% CI)	
		2000–2007	2008–2015			2000–2007	2008–2015
Finland	High	0.4 (0.2–0.7)	0.5 (0.3–0.7)	0.1	0.803	1	1
	Mid	1.0 (0.7–1.3)	1.3 (1.0–1.6)	0.3	0.234	2.5 (1.4–4.4)	2.7 (1.7–4.5)
	Low	3.4 (2.6–4.1)	3.9 (3.0–4.8)	0.5	0.424	8.2 (4.8–14.1)	8.1 (5.0–13.2)
Estonia	High	1.5 (0.8–2.2)	0.4 (0.0–0.7)	− 1.1	0.007	1	1
	Mid	1.5 (0.9–2.1)	0.9 (0.4–1.5)	− 0.6	0.191	1.0 (0.5–2.0)	2.5 (0.9–7.0)
	Low	7.1 (3.8–10.4)	8.4 (4.2–12.6)	1.3	0.646	3.3 (1.7–6.5)	16.2 (5.9–44.4)
Latvia	High	0.2 (0.0–0.4)	0.2 (0.0–0.5)	0.0	0.803	1	1
	Mid	0.8 (0.5–1.2)	0.8 (0.5–1.1)	0.0	0.826	4.0 (1.0–17.0)	3.3 (1.0–11.1)
	Low	5.3 (3.1–7.5)	4.1 (1.9–6.4)	− 1.2	0.465	25.4 (6.0–107.8)	19.2 (5.6–66.0)
Lithuania	High	0.6 (0.2–1.0)	0.5 (0.2–0.9)	− 0.1	0.857	1	1
	Mid	1.1 (0.8–1.4)	0.8 (0.5–1.0)	− 0.3	0.097	1.8 (0.9–3.9)	1.5 (0.7–3.0)
	Low	5.7 (3.3–8.2)	6.1 (3.8–8.3)	0.4	0.826	7.2 (3.2–16.2)	11.7 (5.8–23.9)

*ASMR* age-standardised mortality rate per 100,000 person years, *CI* confidence interval.

Results from the sensitivity analysis showed that excluding register-based records had no effect on female epilepsy mortality in 2000–2015 (Appendix 2). For men, the impact varied by period with a small reduction (− 2.4%) observed in mortality in 2000–2007 while a small increase (+ 1.9%) was seen for the 2008–2015 period.

## Discussion

This study examined educational differences in epilepsy mortality in the Baltic countries and Finland in 2000–2015. Across the period, ASMRs were much higher among men in the Baltic countries compared to those in Finland, whereas for women, ASMRs were similar across the countries. With the exception of women in Finland, there was a reduction in epilepsy ASMRs among both sexes in all countries during the study period. However, this overall reduction masked differences between men and women across the countries according to their level of education. Specifically, there were reductions in epilepsy mortality among high and mid educated men in all of the countries whereas epilepsy mortality increased among low educated men in three of the four countries. This meant that by the end of the study period the educational gradient that was observed in epilepsy mortality rate ratios in 2000–2007 had become more pronounced and ranged between 5 and 10 for low educated men compared to high educated men in all countries. ASMRs were lower for women and there was more variation in the changes that occurred in epilepsy mortality across country educational groups in 2000–2015. Nevertheless, the inverse educational gradient in epilepsy mortality seen in the earlier period still remained in 2008–2015 in all countries but was especially elevated among low educated women in the Baltic countries where epilepsy mortality rate ratios ranged between 11 and 19.

Comparing our findings with those from other studies in the general population is complicated by a range of issues such as differences in the way epilepsy mortality has been classified^16^, the (non-)stratification of the population by sex^24^, as well as the use of data from different time periods^25^. However, there is some indication that epilepsy ASMRs may be elevated among men in the Baltic countries compared to some other countries^19^. The reduction we observed in epilepsy mortality in nearly every male and female country group across the study period accords with the finding from the Global Burden of Disease Study where overall epilepsy ASMRs across 195 countries and territories fell by 24.5% during the 1990–2016 period. The authors of that study suggested that the reduction might be due to improved access to treatment^1^. It can be speculated that this might have also been a factor in the reduction that we observed. However, previous research from Estonia has also shown that not taking anti-seizure medication (ASM) is common^13^ and is linked to an increased mortality risk among individuals with chronic epilepsy^26^. In Estonia and Latvia most/all of the licensed ASMs are fully reimbursed^12^, thus possibly indicating non-adherence to ASMs which may explain the still very high level of epilepsy mortality among men in the Baltic countries.

There was an inverse association between educational level and epilepsy mortality with higher ASMRs in the mid and especially the low educated as compared to the high educated. Although there has been comparatively little research on socioeconomic inequalities in epilepsy mortality to date, our findings are in line with those from previous studies in England and the United States that have linked socioeconomic disadvantage with higher epilepsy mortality^7,8^. It is possible that various factors might play a role in the increased prevalence of epilepsy mortality among low educated individuals. For example, lower education has been associated with more severe epilepsy as measured by hospitalisation/the frequency of hospitalisations^27,28^, while other research has linked lower area socioeconomic status (SES) to emergency medical service assignments for seizures^29^ and individual socioeconomic disadvantage to poor seizure control^30^. This might be important as seizure frequency and severity have been linked to an increased risk for mortality^31^, specifically, SUDEP^32^. In turn, greater seizure frequency and severity might stem from ASM non-compliance, which is higher in the low educated^33^, and which has also been identified as a separate risk factor for SUDEP^34^, although this finding remains contested^32^. Future research should be undertaken in our study countries to determine the role of SES in ASM non-compliance, and whether lower ASM use is linked to mortality, especially as an earlier study from Estonia showed that a large proportion of PWE (22%) were not taking ASMs, indicating a high treatment gap^35^.

The inverse educational gradient observed in 2000–2007 widened in 2008–2015 with epilepsy mortality increasing among low educated men and women in all countries except Latvia, while it either fell or remained the same for the mid and high educated in all of the countries except among Finnish women. These changes coincided with the onset of the Great Recession in 2008–2009. At that time there was a sharp rise in overall unemployment (especially in the Baltic countries)^22^, although there is some indication that the low educated may have been disproportionately affected across time^36^. In addition, for those who remained in work government austerity measures in response to the economic crisis resulted in reduced social benefits as well as wage cuts for public sector workers^37^. In this context, perceived financial distress might have been important for mortality as it has been linked to ASM non-compliance in other research^38^. It is also possible that educational differences with respect to access to specialist epilepsy care that have been observed in other settings^27,39^ might have been exacerbated in this period although we observed a sharp fall in epilepsy ASMRs among the low educated in Latvia, where there is some evidence that unmet medical need grew sharply in 2010–2012 due to an inability to afford medical care^40^.

This study has a number of strengths including being able to use across-time population-based register data for four countries to undertake one of the first examinations of the role of socioeconomic differences in epilepsy mortality in the general population. However, the study also has several limitations. First, it should be noted that although we focused on epilepsy as an underlying cause of death, these deaths constitute only a minority of all deaths among PWE. An earlier study that reported on epilepsy mortality in England in 2001–2006 found that epilepsy was recorded as the underlying cause of death on only 48% of the death certificates where epilepsy was mentioned^19^. Another study in a well-defined cohort of PWE indicated that even fewer deaths may be recorded as only 7% of death certificates mentioned epilepsy^41^. Indeed, a major issue for using death certificates to identify SUDEP is that it is not a recognised category in ICD-10^42^. Thus, studies that rely on epilepsy as an underlying cause of death will inevitably underestimate epilepsy mortality; however, we do not have any evidence that underestimation is related to specific educational groups. Second, we were not able to control for the potentially confounding effects of early-onset epilepsy and/or epilepsy severity in this study that may have impacted educational attainment. Third, it is possible that other measures of SES besides education, such as wealth/poverty, may have also been important for the observed associations but we lacked the data to examine the independent effects of these factors. Fourth, the small number of epilepsy deaths, especially among the high educated and among women, resulted in large confidence intervals for some of the estimates, and may partly explain the sometimes very large rate ratios measuring educational inequalities. Fifth, although a sensitivity analysis using Latvian data showed that excluding register-only-based records had little impact on overall epilepsy mortality, we cannot discount the possibility that the effect of excluding these data might have varied between different educational groups, which could have affected our results. Finally, given the comparative lack of epidemiological data on epilepsy in the Baltic countries^12^, it was not possible to examine our results in terms of factors such as ASM access across the period or the incidence of epilepsy^24^ that might have helped us to better contextualise our findings. This highlights the urgent need for more epilepsy-related research to be undertaken in the Baltic countries, especially among those with low SES.

In conclusion, this study shows that there are differences in epilepsy mortality in the Baltic countries and Finland by education level and that ASMRs are elevated among men in the Baltic countries compared to their counterparts in Finland. Importantly, inequalities in epilepsy mortality grew among men and women (except among Latvian females) in all of the countries across the study period, highlighting the need for action to reduce the comparatively high epilepsy mortality rates amongst lower educated individuals.

## Supplementary Information

Supplementary Information.

## Data Availability

The data that support the findings of this study are available from National Statistical Offices, i.e. Statistics Estonia, Statistics Lithuania, Central Statistical Bureau of Latvia and Statistics Finland but restrictions apply on the availability of these data, which were used under license for the current study, and so are not publicly available. Data are however available from the authors upon reasonable request and with the permission of the data providers.
