# Supplementary material for: Educational inequalities in epilepsy mortality in the Baltic countries and Finland in 2000–2015
Source: Sci Rep. 2022 Mar 17;12:4597. doi: 10.1038/s41598-022-08456-x (PMC8930999; doi:10.1038/s41598-022-08456-x)
Supplement: Supplementary file 1 — Supplementary Information. [file 41598_2022_8456_MOESM1_ESM.docx]

**Supplementary information**

Educational inequalities in epilepsy mortality in the Baltic countries and Finland in 2000–2015.

Andrew Stickley, Aidan Neligan, Aleksei Baburin, Domantas Jasilionis, Juris Krumins, Pekka Martikainen, Naoki Kondo, Tomiki Sumiyoshi, Jae Il Shin, Hans Oh, Kyle Waldman, Mall Leinsalu

**Appendix 1** Causes of Death ICD-10: G40, G41^[[1]](#footnote-1)^

| **Cause of death** |
| --- |
| G40 Epilepsy |
| G40.0 Localization-related (focal)(partial) idiopathic epilepsy and epileptic syndromes with seizures of localized onset |
| G40.1 Localization-related (focal)(partial) symptomatic epilepsy and epileptic syndromes with simple partial seizures |
| G40.2 Localization-related (focal)(partial) symptomatic epilepsy and epileptic syndromes with complex partial seizures |
| G40.3 Generalized idiopathic epilepsy and epileptic syndromes |
| G40.4 Other generalized epilepsy and epileptic syndromes |
| G40.5 Special epileptic syndromes |
| G40.6 Grand mal seizures, unspecified (with or without petit mal) |
| G40.7 Petit mal, unspecified, without grand mal seizures |
| G40.8 Other epilepsy |
| G40.9 Epilepsy, unspecified |
| G41 Status epilepticus |
| G41.0 Grand mal status epilepticus |
| G41.1 Petit mal status epilepticus |
| G41.2 Complex partial status epilepticus |
| G41.8 Other status epilepticus |
| G41.9 Status epilepticus, unspecified |

**Appendix 2** Impact of excluding register-only-based census records on epilepsy mortality among 30–74 year olds in Latvia, 2000–2015

| Period | Men |  |  | Women |  |  |
| --- | --- | --- | --- | --- | --- | --- |
|  | Census + registry | Census |  | Census + registry | Census |  |
|  | ASMR (95% CI) | ASMR (95% CI) | Difference, % | ASMR (95% CI) | ASMR (95% CI) | Difference, % |
|  |  |  |  |  |  |  |
| 2000–2007 | 8.6 (7.7–9.5) | 8.4 (7.5–9.3) | -2.4 | 1.2 (0.9–1.5) | 1.2 (0.9–1.6) | 0 |
| 2008–2015 | 5.3 (4.6–6.0) | 5.4 (4.7–6.1) | 1.9 | 1.0 (0.7–1.3) | 1.0 (0.7–1.3) | 0 |
|  |  |  |  |  |  |  |
| ASMR, age-standardized mortality rate per 100 000 person years; CI, confidence interval. | | | | |  |  |

1. World Health Organization. International Classification of Diseases and Related Health Problems (10th Revision, Volume 1) Tabular list. Fifth Edition. Geneva: WHO; 2016. [↑](#footnote-ref-1)
